# The Community PrEP Study: a randomized control trial leveraging community-based platforms to improve access and adherence to pre-exposure prophylaxis to prevent HIV among adolescent girls and young women in South Africa—study protocol

**DOI:** 10.1186/s13063-021-05402-6

**Published:** 2021-07-26

**Authors:** Andrew Medina-Marino, Dana Bezuidenhout, Sybil Hosek, Ruanne V. Barnabas, Millicent Atujuna, Charl Bezuidenhout, Phuti Ngwepe, Remco P.H. Peters, Francesca Little, Connie L. Celum, Joseph Daniels, Linda-Gail Bekker

**Affiliations:** 1 Research Unit, Foundation for Professional Development, 10 Rochester Rd, Vincent, East London, Buffalo City Metro, Eastern Cape Province South Africa; 2 The Desmond Tutu HIV Centre, University of Cape Town, Anzio Road, Observatory, Cape Town, South Africa; 3 Perelman School of Medicine, University of Pennsylvania, Philadelphia, PA USA; 4 Division of Child and Adolescent Psychiatry, Department of Psychiatry, Stroger Hospital of Cook County, Chicago, IL USA; 5 Department of Global Health, University of Washington, Seattle, WA USA; 6 Department of Epidemiology, University of Washington, Seattle, WA USA; 7 Department of Statistical Sciences, University of Cape Town, Cape Town, South Africa; 8 Department of Psychiatry and Human Behaviors, Charles R. Drew University of Medicine and Science, Los Angeles, USA

**Keywords:** HIV, HIV prevention, PrEP, Randomized controlled trial, Adolescent girls and young women, Community-based delivery platforms, Adherence support intervention, Cost-effectiveness, South Africa

## Abstract

**Background:**

HIV incidence among South African adolescent girls and young women (AGYW) remains high, but could be reduced by highly effective pre-exposure prophylaxis (PrEP). Unfortunately, AGYW report significant barriers to clinic-based sexual and reproductive health services. Even when AGYW access PrEP as an HIV prevention method, poor prevention-effective use was a serious barrier to achieving its optimal HIV prevention benefits. Determining the acceptability and feasibility of community-based platforms to increase AGYW’s access to PrEP, and evaluating behavioural interventions to improve prevention-effective use of PrEP are needed.

**Methods:**

We propose a mixed-methods study among AGYW aged 16–25 years in Eastern Cape Province, South Africa. In the first component, a cross-sectional study will assess the acceptability and feasibility of leveraging community-based HIV counselling and testing (CBCT) platforms to refer HIV-negative, at-risk AGYW to non-clinic-based, same-day PrEP initiation services. In the second component, we will enrol 480 AGYW initiating PrEP via our CBCT platforms into a three-armed (1:1:1) randomized control trial (RCT) that will evaluate the effectiveness of adherence support interventions to improve the prevention-effective use of PrEP. Adherence will be measured over 24 months via tenofovir-diphosphate blood concentration levels. Qualitative investigations will explore participant, staff, and community experiences associated with community-based PrEP services, adherence support activities, study implementation, and community awareness. Costs and scalability of service platforms and interventions will be evaluated.

**Discussion:**

This will be the first study to assess the acceptability and feasibility of leveraging CBCT platforms to identify and refer at-risk AGYW to community-based, same-day PrEP initiation services. It will also provide quantitative and qualitative results to inform adherence support activities and services that promote the prevention-effective use of PrEP among AGYW. By applying principles of implementation science, behavioural science, and health economics research, we aim to inform strategies to improve access to and prevention-effective use of PrEP by AGYW.

**Trial registration:**

ClinicalTrials.govNCT03977181. Registered on 6 June 2019—retrospectively registered.

## Background

In 2017, 1.8 million people were infected with human immunodeficiency virus (HIV), most of whom live in Southern and Eastern Africa [1]. There exist significant differences in HIV incidence and prevalence by age and sex, with young women being particularly vulnerable to HIV [2–7]. In South Africa, specifically, HIV incidence and prevalence among adolescent girls and young women (AGYW) ages 15–24 is significantly higher than their male counterparts (incidence = 1.51% per year vs. 0.49% per year, respectively; prevalence = 10.9% vs 4.8%, respectively) [8]. Significant risk factors include having concurrent or older sexual partners [9–11] as well as interpersonal violence [12, 13]. Consequently, there is an urgent need for effective HIV prevention strategies targeted at young women and under their control, to reduce HIV incidence among this key population.

Pre-exposure prophylaxis (PrEP) with emtricitabine (FTC)/tenofovir (TDF) as a method of HIV prevention has demonstrated significant efficacy in clinical trials among different population groups, with efficacy being strongly associated with increased drug adherence [14–18]. Previous and current PrEP demonstration projects have largely focused on providing PrEP through clinic-based delivery platforms, including antiretroviral therapy (ART), sexually transmitted infections (STI), family planning, and adolescent-friendly clinics [19–21]. While clinic-based services may provide good opportunity for service integration, leveraging these facility-based services for PrEP provision may be fraught with barriers including perceived lack of confidentiality and privacy at health centres [22–24], unfriendly clinic staff [25, 26], long queues, inconvenient operating hours, and a perceived predominant focus on maternal-child health with no AGYW-focused services [27, 28].

Interventions associated with adolescent health should be adolescent-centred, with services leveraging community-based delivery platforms and not rigid, medicalized, clinic-based venues [29]. Recent findings from an at-scale community-based HIV counselling and testing (CBCT) programme implemented in South Africa found that AGYW had the highest testing uptake across all age groups of either sex, regardless of district type (i.e. urban, rural, peri-urban) [30]. Given that HIV testing is the entry point for PrEP service delivery, leveraging CBCT platforms to effectively identify and link young women to PrEP services should be considered as a supply-side intervention for scaling up PrEP.

Community-based programmes aimed at supporting ART adherence and retention in care have been recognized as effective and sustainable approaches to achieving the UNAIDS 90-90-90 targets [31–37]. One of the most innovative and successful adherence support interventions has been “adherence clubs”. Adherence clubs are a cost-effective intervention strategy that can be locally adapted to low-resource settings and implemented by non-specialists and lay health workers with little formal counselling experience [37–40]. As PrEP becomes more widely available, rigorously evaluated adherence skill-building interventions, implemented through community-based platforms, will provide invaluable information regarding the optimal structure, content, and delivery of PrEP adherence support programmes.

## Study rationale and aims

Few studies have explored effective strategies to identify and deliver non-clinic-based PrEP services to at-risk populations, how to support prevention-effective use and retention in PrEP care, or implementation science approaches to scaling up PrEP delivery. Given that CBCT platforms have been shown to be highly acceptable and accessible to adolescent girls and young women, it is crucial to understand if these platforms can also be used to identify and deliver PrEP to at-risk youth [14–16]. Furthermore, community-based prevention-effective PrEP adherence support is critical to reduce the HIV burden among AGYW in South Africa. Towards this, our study aims include (Table 1):

- Aim 1: PrEP service delivery: To determine the acceptability and feasibility of PrEP uptake by AGYW when delivered through community-based HIV counselling and testing platforms;
- Aim 2: PrEP adherence support: To evaluate community-based, scalable interventions to achieve prevention-effective adherence to PrEP among AGYW; and
- Aim 3: Scalability of community-based PrEP services: estimate the cost per AGYW initiated on PrEP and provided adherence support through community-based platforms, and the cost per incident HIV infection averted.

**Table 1:** Study overview: aims, rational, hypothesis, design and outcomes

	Hypothesis	Rational	Study design and approach	Primary outcome	Secondary outcomes
**Aim 1**	CBCT platforms will facilitate access to and initiation of PrEP by adolescent girls and young women	Numerous barriers exist that delay or inhibit YW’s access to clinic-based reproductive health and HIV prevention services	Cross-sectional, mixed methods. Integrate PrEP initiation services into CBCT pop-up testing sites and home-based testing platforms in extremely high HIV burden and under-researched rural and urban settings of South Africa	The proportion of eligible young women accepting PrEP as part of CBCT services	(1) PrEP initiation rates by a) community type (urban vs. rural), and b) CBCT platform (*pop-up testing sites vs systematic home-based testing*); (2) In-depth qualitative understanding of immediate, delayed and never accepting PrEP; (3) Socio-demographic and behavioural correlates of immediate, delayed and never initiating PrEP
**Aim 2**	Cognitive behavioural-based adherence support programmes will be associated with a substantial increase in prevention-effective use of PrEP compared to a control	Lack of PrEP efficacy in African YW in clinical trials was strongly associated with poor adherence. To maximize the prevention benefits of PrEP, scalable adherence support interventions are needed	Randomized controlled trial. Enrol and randomize *PrEP-accepting young women* to receive one of two adherence support interventions or standard care (basic adherence support)	Comparison of tenofovir-diphosphate concentration levels in dried blood spots by study arm, with a 12-month primary outcome and 24-month assessment of intervention durability	(1) Predictors of PrEP adherence after adjusting for study arm, socio-demographic factors, exposure to adherence support activities, and risk profiles; (2) Characterization of changes in sexual behaviours and risk profiles following PrEP initiation; (3) Proportion of individuals that discontinue PrEP and factors associated with discontinuation; and (4) Qualitative description and factors associated with of patterns of PrEP use
**Aim 3**	Community-based PrEP initiation and adherence support platforms will increase uptake of and adherence to PrEP among YW, and cost-effectively avert HIV infections compared to standard practice	Evidence on cost and cost-effectiveness can be used by decision makers regarding the use of resources to support PrEP scale-up	Mathematical modelling and micro-costing studies. Estimate incremental costs and cost-effectiveness of community-based PrEP initiation and adherence support programmes among YW relative to standard practice.	Estimate the cost-effectiveness and budget impact of targeted PrEP initiation and adherence support	(1) Micro-costing studies will evaluate costs incurred and costs averted; (2) Estimate impact of support interventions on HIV incidence, HIV-related deaths and DALYs, using proportion on PrEP

## Methods

### Study design and flow

The Community PrEP Study will enrol a cohort of 480 HIV-negative South African AGYW aged 16–25 years, incorporating multiple study designs to address our three main study aims (Table 1). Specifically, a cross-sectional, mixed-methods study design will be used to quantitatively measure and qualitatively assess the acceptability and feasibility of using CBCT platforms to identify HIV-uninfected AGYW and offer PrEP (Aim 1). A randomized controlled trial will evaluate a behavioural intervention aimed at supporting prevention-effective adherence to PrEP (Aim 2). Finally, we will perform micro-costing and mathematical modelling analyses to calculate the cost-effectiveness of our PrEP delivery platform and adherence support programmes (Aim 3). Community advisory boards (CABs) were established to facilitate community engagement and acceptance, elicit feedback on study design, conduct analysis, and ensure that community views and perspectives were appropriately incorporated in a collaborative manner.

### Study location

**Fig. 1:**
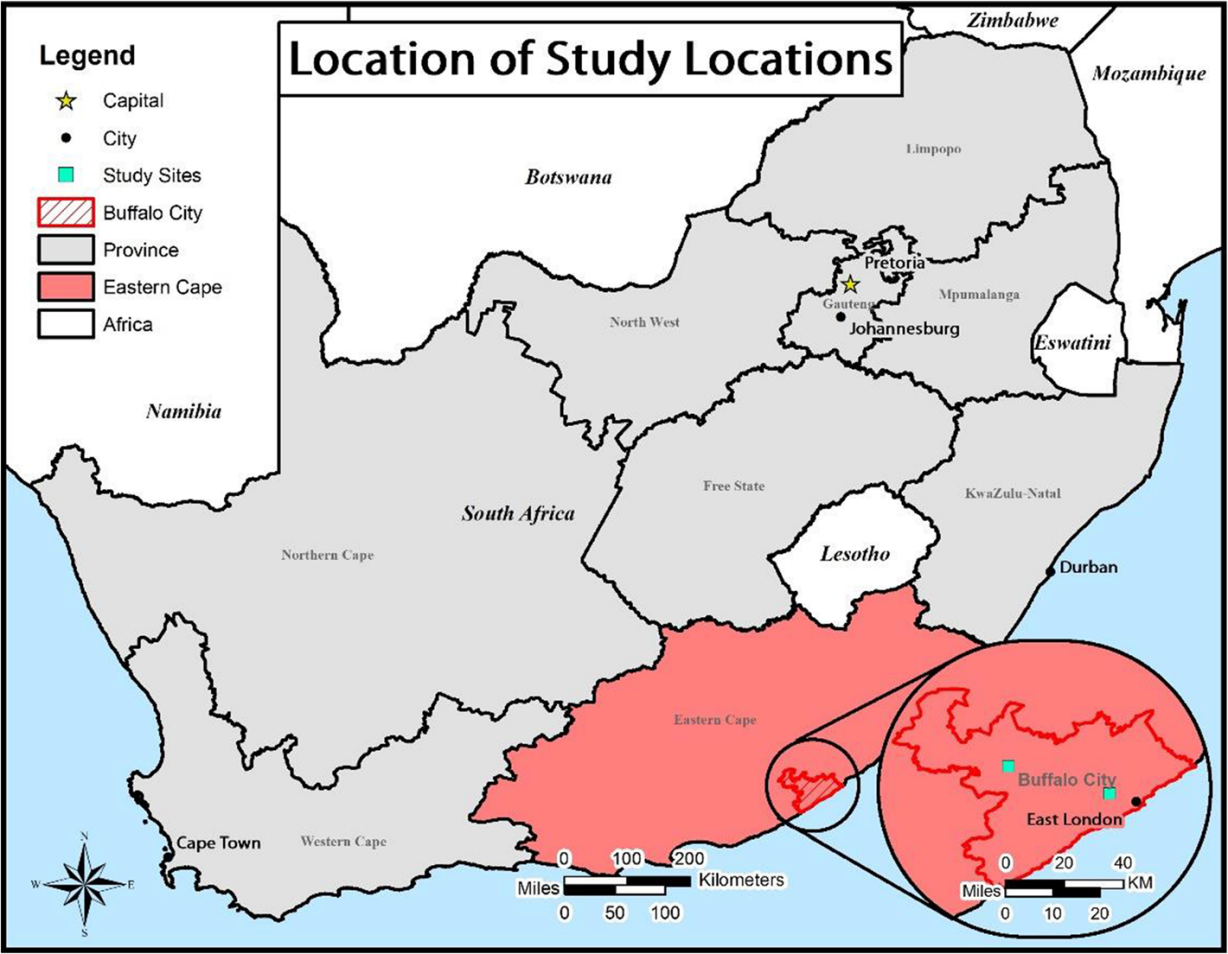
Provincial map of South Africa showing location of study communities within Buffalo City Metro Health District, Eastern Cape Province

This study is being conducted in Buffalo City Metro (BCM) Health District, Eastern Cape Province, South Africa (Fig. 1). In 2016, BCM had an estimated mid-year population of 834,998 people. In 2016, the South African National Department of Health estimated that BCM had a general population HIV prevalence of 12.4% and incidence of 0.54%; an adult (15+ years old) HIV prevalence of 17.1% and incidence of 0.74%; and a female youth (age 15–24 years) HIV prevalence of 12.8% and incidence of 2.40% [41]. Within BCM, our study will be conducted in one urban and one rural community. Selection of study communities was conducted in consultation with the BCM Department of health and was informed by census data, absence of other ongoing HIV prevention studies, and community willingness to be involved in the study.

### Intervention design and adherence support activities

#### Description of the intervention

The intervention arms were developed using the Information-Motivation-Behavioural Skills Model of Behaviour Change (IMB) [42, 43]; the IMB model has been used extensively to improve medication adherence, including for ART [42–44] and daily contraceptives [45]. Intervention arms will incorporate content from the evidence-based intervention Life Steps [46], as well as from the HIV Prevention Trials Network 082 study [47] and the 3Ps for Prevention open-label PrEP demonstration study [48]. Using standardized manuals, adherence support interventions will be delivered in either a group (Arm 1) or one-on-one individual (Arm 2) sessions. Participants randomized to intervention arms will be seen by adherence counsellors monthly for 24 months at a fixed community-based site.

#### Control

Control participants will receive passive adherence support consistent with standard of care for PrEP programmes delivered by public sector health clinics. Control participants will return monthly to collect medication refills, report and receive support for side effects, and conduct special study activities (i.e. specimen collection; Table 2).

**Table 2:** Study related events and time points

	Baseline	M1	M2	M3	M6	M9	M12	M15	M18	M21	M24
**Routine study activities**											
**HIV and pregnancy testing**	**X**			**X**	**X**	**X**	**X**	**X**	**X**	**X**	**X**
**Group and individual adherence support**		**X**	**X**	**X**	**X**	**X**	**X**	**X**	**X**	**X**	**X**
**PrEP medication pickup**	**X**	**X**	**X**	**X**	**X**	**X**	**X**	**X**	**X**	**X**	
Special study activities*											
**ACASI Questionnaire**	**X**			**X**			**X**				**X**
**CrCl testing**	**X**			**X**			**X**				**X**
**STI testing**	**X**				**X**		**X**				**X**
**Blood testing for detectable TFV-DP**				**X**	**X**		**X**		**X**		**X**
**Hepatitis B testing and vaccination**	**X**	**X**			**X**						

* A R30.00 (~$2.00) voucher will be provided after each “special” study visit where specimens are collected (i.e. M3, 6, 12, 18, and 24)

Monthly activities will include (1) attendance check-in, (2) adherence support session (intervention arms only), (3) clinical assessment, (4) medication dispensing, (5) study visit reminders, and (6) study pack and voucher. Each month, study staff will contact participants up to three times via SMS and phone calls as study visit reminders to support retention. More details about intervention and control arms is available in Table 3.

**Table 3:**
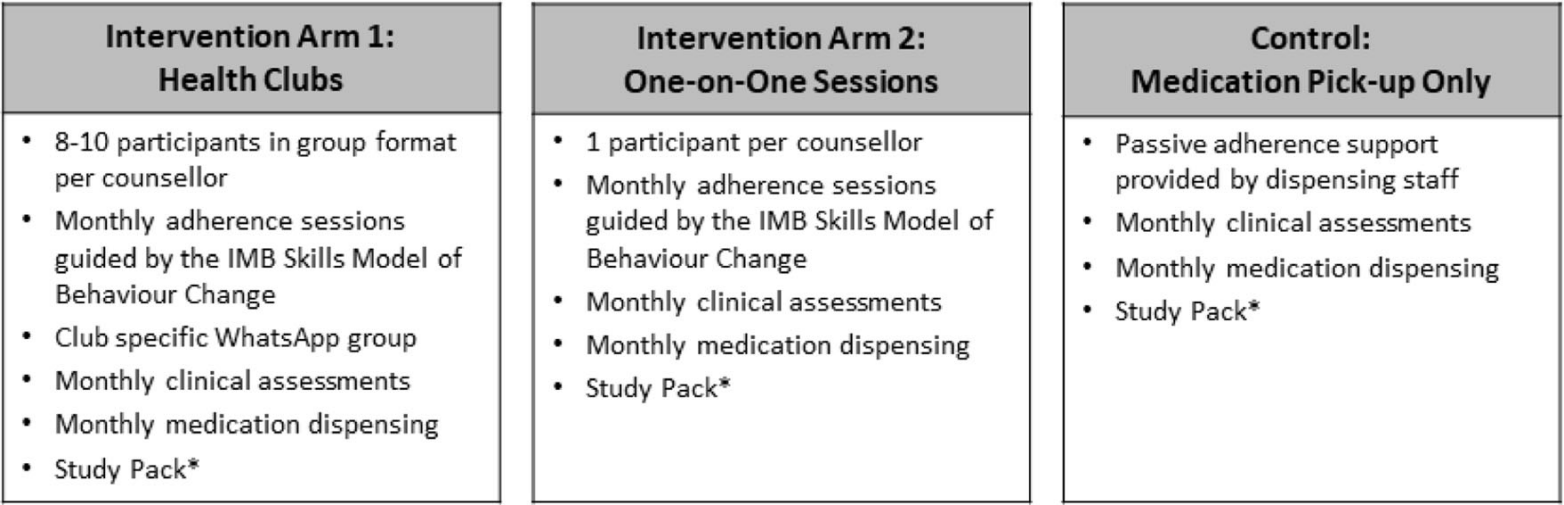
Components of adherence interventions

*Study packs included sanitary pads, condoms, snack, and medication refill reminder card

### Randomization

Randomization will occur following participant consent and confirmatory pregnancy testing. Independent random allocation lists will be created by the study Data Manager for each study site (urban vs. rural), with study staff and participants blinded to the allocation scheme. Participants will be randomized to study arms in a 1:1:1 ratio using computer-generated quasi-random numbers and permuted blocks of 18, within the randomization module of Research Electronic Data Capture (REDCap) [49]. Since the randomization occurs at the community-based study sites, it is impossible to blind study team members or participants to the randomization assignments. However, procedures to minimize the influence of the unblinded nature of this study on outcomes have been implemented. Ongoing data monitoring does not include information about study endpoints disaggregated by study site or study arm. Although the study biostatistician reviews data on PrEP uptake and adherence by study arm and site, the final outcome is always blinded.

### Recruitment

Participants will be recruited from community-based HIV counselling and testing platforms established in study communities for the purpose of this study. Consent and enrolment will be conducted by field-based enrolment counsellors.

#### HIV testing and testing platforms

Two different CBCT modalities, *pop-up site* and *home-based testing*, will be leveraged in each community. HIV testing will be conducted in accordance with South African national guidelines [50]. Referrals to local HIV treatment services will be provided to those with a confirmed positive result.

##### Pop-up testing sites

Three pop-up testing sites will be leveraged in each study community. The main site (Site A) will be a fixed-positioned testing venue co-located with a centralized community venue for the full duration of the study. A second site (Site B) will be a semi-mobile container co-located with another recognizable community venue during enrolment. A third site (Site C) will be a highly mobile testing tent placed at peripheral locations in each community, and repositioned 2–3 times during enrolment.

##### Door-to-door home-based testing

Prior to initiating home-based testing activities, GIS maps will be obtained for each community, with 24 urban and 26 rural small-block areas delineated and individual houses plotted for targeted testing. Detailed maps and electronic tablets will be used to track the small-block areas and houses visited and tested. Home-based testing will be performed until all houses are visited at least once.

#### PrEP initiation and study enrolment

##### Baseline screening for referral for PrEP study and services

Prior to screening, participants will be asked to provide fingerprints to facilitate participant identification during subsequent study activities; fingerprints will be collected using the FS88 Futronic Fingerprint Scanner [Futronic Technology Company, Kwai Fong, Hong Kong]. Participants will then be asked to complete an audio computer-assisted self-interviewing (ACASI) questionnaire collecting socio-demographic, behavioural, and clinical histories; HIV knowledge, attitudes, and practice; and a self-reported HIV risk survey. Upon completing the ACASI, participants will watch a PrEP informational video [51], followed by an interviewer-administered screening tool to assess whether the participant is currently (1) interested in learning more about PrEP, (2) interested in taking PrEP, (3) using post-exposure prophylaxis (PEP), (4) participating in other HIV prevention studies, (5) pregnant, (6) breast feeding, (7) on TB treatment, or (8) has signs or symptoms of acute HIV infection. Responses will be captured in real time using REDCap.

##### Inclusion/exclusion criteria

A candidate is eligible for referral to PrEP initiation services if they (1) are aged 16–25 years; (2) self-identify as female; (3) have a HIV-negative test result obtained from a study-associated CBCT site; (4) plan on residing in the study community for at least 12 months; (5) complete the baseline questionnaire; (6) express interest in taking PrEP; (7) not taking TB treatment; (8) not pregnant or breastfeeding; (9) not currently using PEP; (10) not currently participating in other HIV prevention studies; (11) do not have signs or symptoms of acute HIV infection; (12) understand English or IsiXhosa. Of note, given the likelihood of social desirability bias, structural risk factors, and the dynamic nature of HIV risk among South African AGYW, prior sexual activity will not be used as inclusion/exclusion criterion [8, 52, 53].

##### Referral for PrEP services and study enrolment

Eligible participants interested in PrEP will be provided a copy of their HIV test results and a referral letter for PrEP initiation at our study sites, which will be co-located with pop-up testing sites A and B. Upon presenting to a PrEP study site, participants will be consented, asked to provide contact details, tested for syphilis and pregnancy, and asked to submit a self-collected vaginal swab for further STI testing (Table 2). If pregnant, participants will be provided a referral letter for antenatal care services in lieu of PrEP initiation, as South Africa has not yet approved PrEP initiation during pregnancy. Prior to departure, participants will receive their syphilis test results, with a treatment referral if applicable.

Participants will be asked to provide a blood sample for hepatitis B testing and creatinine clearance, randomized, and then provided a 30-day supply (1 bottle) of DIDIVIR (CIPLA generic for Emtricitabine 200 mg/ Tenofovir Disoproxil Fumarate 300 mg); those randomized to an intervention group will be reminded of their first adherence session date. All participants will be asked to return for an orientation visit 2 weeks post-enrolment to meet with their adherence counsellor, and a study nurse to review any test results, assess side effects and acute HIV symptoms, and receive the first of three hepatitis B injections if indicated.

### Data collection, management, and oversight

A mixed-methods approach will be used to collect data from participants and study staff throughout the 2-year study follow-up period.

#### Quantitative data

*Quantitative data* will be captured at baseline and again at months 3, 12, and 24 via ACASI, about participants’ sexual behaviour, risk factors, self-perception of HIV acquisition risk, partner characteristics and HIV status, knowledge and attitudes towards PrEP, perceived barriers and facilitators of PrEP uptake and adherence, projected HIV stigma, alcohol and drug use, mental health, and social support [54–57]. Basic adherence tracking, specimen collection, test results, and participant timelines will be monitored on REDCap.

#### Qualitative data

*Qualitative data* (Table 4) will be collected using in-depth interviews (IDIs) and community mapping exercises (CMEs) with AGYW, focus group discussions (FGDs) with community advisory board members, and staff exchange programmes. All IDI and FGD guides will be semi-structured, and studies will be conducted in the preferred language of the participant.

**Table 4:** Qualitative data collection schedule

Category	Case definition	Data collection method	Participant type	# of interviews
**Special circumstances among study participants**				
Seroconverts	Participant tests HIV positive during any follow visit. HIV test confirmed with ELISA test.	IDI	PrEP Participant	3 IDIs
Discontinued PrEP	Participants who decide to discontinue PrEP during any time of the study follow-up period.	IDI	PrEP Participant	9 IDIs (3 per arm)
Two or more consecutive missed visits	Participants who missed two or more consecutive adherence visits during follow-up period.	IDI	PrEP Participant	12 IDIs
Reported social harms	Participants who report any type of social harm due to study participation. i.e. participants treated unfairly and experiencing problems being accepted by their families, partners, and/or communities due to participation in the study	IDI	PrEP Participant	All Cases of Social Harms
Unique pattern of medication use	Participants who have a unique pattern of medication use as identified by adherence counsellors or study nurses. i.e. Use over holidays and/or over weekends only	IDI	PrEP Participant	9 IDIs (3 per arm)
Never presented for medication refill following enrollment	Participants who never presented to study sites for adherence sessions/medication collection after initiating PrEP.	IDI	PrEP Participant	15 IDIs
**PrEP uptake from community platforms**				
Immediate acceptors	Participants who consent to study and receive supply of PrEP the day of screening and study introduction.	IDI	Pop-up Site Testing Enrolment	15 IDIs
Immediate presenters	Participants who present to study sites for PrEP initiation within 0-3 days of completing baseline questionnaire.	IDI	Home-based Testing Enrolment	15 IDIs
Early presenters	Participants who present to study sites for PrEP initiation within 4-14 days of completing baseline questionnaire.	IDI	Home-based Testing Enrolment	15 IDIs
Late presenters	Participants who present to study sites for PrEP initiation within 15-30 days of completing baseline questionnaire.	IDI	Home-based Testing Enrolment	15 IDIs
Never presenters	Participants who never presented to study sites after completing baseline questionnaire.	IDI	Home-based Testing Enrolment	15 IDIs
**Experiences with taking PrEP and engaging in adherence support activities**				
High adherence	Active participants who have attended more than one adherence session with DBS levels ≥ 700 fmol/punch.	IDI and CME	PrEP Participants	20 IDIs (5 per intervention arm per site)
Low adherence	Active participants who have attended more than one adherence session with DBS levels ≤ 699 fmol/punch.	IDI and CME	PrEP Participants	20 IDIs (5 per intervention arm per site)
**Process evaluation and staff implementation experiences**				
PrEP field staff	Interviews with field staff, including research nurses, adherence counsellors, and field workers to understand their experiences with PrEP implementation at a community-based level.	IDI	Field Staff	12 IDIs (6 per site)
Observational exchange exercise	Exchange of one staff member per site to observe differences in staff interaction with PrEP participants that may influence participant adherence and attendance.	Staff Exchange	Field Staff	1 day per site
**Community preparation and engagement**				
CAB members	Active community advisory members who advised study staff on study implementation, study progress, and community engagement.	FGD	CAB Members	4 FGDs (2 per site)

#### AGYW interviews

*AGYW interviews* will be used to understand their decisions on whether or not to access PrEP services, timing of PrEP initiation, early product experience, and disclosure challenges. A second cadre of interviews will focus on motivation of continued PrEP use and factors influencing adherence. During these interviews, participants will complete two mapping activities: (1) community map of their neighbourhoods to outline social settings influencing PrEP uptake and adherence; and (2) map of their home to describe resources (water/ food security, privacy etc.) and family factors influencing PrEP management and dosing plans. A third group of interviews will explore special circumstances occurring among study participants.

#### Community advisory board (CAB) and staff exchange programmes (SEPs)

We will conduct FGDs to assess CAB experiences and perspectives of community acceptability of PrEP for AGYW. A SEP using observations and staff IDIs will allow those doing the same work at different sites to recognize and discuss implementation differences that may inform optimized service delivery and participant engagement. FGDs and SEPs will assess community and study impact on adherence or attendance, as well as determining additional differences in community and organization that may influence these study outcomes.

#### Data management and quality assurance

Data will be stored in a secure, password-protected, web-based database which will only be accessible to authorized project staff. Tablet computers used for ACASI interviews will be password protected and will be stored securely at the Foundation for Professional Development (FPD) offices in the study districts. Paper records of participants will be kept in lockable filing cabinets at FPD offices. Paper records, excluding informed consent forms, will only contain PINs. A separate, access-controlled, link log database will be developed and maintained by the data manager. Qualitative data including audio files and password-protected transcripts will be stored on a secure, access-controlled cloud-based database (SharePoint). Double-data entry of laboratory results will be employed when capturing participant data onto RedCap. Automated data quality checks and skip patterns will be built into RedCap and ACASI. Research assistants will conduct onsite data quality checks daily. Scheduled and unscheduled data quality inspections will be carried out by the data quality assurance personnel in order to ensure high data quality standards. The principal investigators, or their designate, will randomly select 10% of all participant files for inspection every 3 months.

#### Study oversite, data safety and monitoring board (DSMB), and study auditing

The co-principal investigators/corresponding authors take responsibility for the scientific validity of the study protocol, assessment of study quality and conduct, as well as for the scientific quality of the final study report. The primary author oversees the coordinating centre and takes responsibility for the day-to-day study implementation in collaboration with three other co-authors (Foundation for Professional Development). A DSMB will be made up of four invited national and international experts, at least two of whom are clinically trained and knowledgeable about PrEP and at least one biostatistician. DSMB members will have no direct association with this study and will be independent from any professional or financial conflict of interest with the research project and/or study investigators. The DSMB will review in aggregate on a bi-annual basis, any adverse events (AEs) that occur. Though no serious adverse events (SAEs) are reasonably expected during this trial, should they occur the DSMB will review them in real time. Trial conduct will be continuously audited as part of quarterly meetings of the study team, and annually by the University of Cape Town Human Research Ethics Committee as part of mandatory renewal of ethics approval.

### Laboratory testing, STI treatment, and clinical follow-up

#### HIV rapid testing

HIV counselling and testing will be performed every 3 months, per South African national guidelines [50] (Table 2). Those with a positive confirmatory test will have venous blood drawn to test for HIV drug resistance. Furthermore, those testing positive for HIV will be terminated from the study and provided a referral letter for HIV care and treatment.

#### STI testing and treatment

We will test for *Chlamydia trachomatis* (CT), *Neisseria gonorrhoeae* (NG), and *Trichomonas vaginalis* (TV) using a self-collected vaginal swab at baseline and again at months 6, 12, and 24. Self-collected swabs will be shipped monthly to the National Institute for Communicable Diseases (NICD; Johannesburg, South Africa) for nucleic acid amplification testing (NAAT) using the Aptima Combo 2® Assay (Hologic, San Diego, USA) for *Neisseria gonorrhoeae* and *Chlamydia trachomatis* and using the Aptima® Trichomonas vaginalis Assay (Hologic, San Diego, USA) for *Trichomonas vaginalis.* Negative test results will be notified at the next study visit. Positive test results will be notified immediately upon result availability via site-based visits or phone calls. Those with a positive STI test result will be provided a copy of their results and a treatment referral letter per South African STI guidelines [58].

Syphilis testing will be conducted during the enrolment visit only, using the Alere Determine™ Syphilis TP rapid test. Negative tests will be reported by the study nurse to participants; patients testing positive will be referred for further laboratory confirmatory testing and treatment as indicated. All enrolled participants will be tested for Hepatitis B antigen (HBsAg), performed by the National Health Laboratory Service. Participants with a negative test result will be provided hepatitis B vaccination per the South African vaccination schedule [59]; those that test positive will be asked to provide blood for ALT and AST testing to determine liver function and referred for care and treatment. Neither the syphilis nor hepatitis B test results will impact study eligibility.

#### Pregnancy testing

Urine-based pregnancy testing to detect human chorionic gonadotropin will be conducted every 3 months. If a participant becomes pregnant while enrolled in the study, they will be provided referral letters for prenatal support and care, and counselled on taking PrEP while pregnant, and will be allowed to decide whether they want to continue PrEP. Participants who continue taking PrEP will be monitored by the study clinician and nurses, with pregnancy milestone and birth outcomes recorded and monitored.

#### Creatinine testing

Creatinine clearance will be assessed to monitor kidney function at baseline, month 3, month 12, and month 24. Clinical assessment for PrEP continuation will occur if estimated glomerular filtration (eGRF) rate is less than 60 mL/min. If eGRF does not improve, the participant will be terminated from the study due to risk of kidney damage and will be provided a referral letters for renal management and care.

#### Tenofovir testing

To assess PrEP adherence, tenofovir-diphosphate levels will be assessed at months 3, 6, 12, 18, and 24. Dried blood spots (DBS) will be prepared from venous blood and spotted on Whatman protein saver cards. Drug concentration testing will occur at the University of Cape Town Division of Pharmacology laboratory using DBS TDF testing protocols [60].

### Sample size and power calculations

A sample size was calculated to have 80% power to detect differences in the proportion of participants adhering to PrEP among the three study arms (Aim 2) using the chi-square test for independence with two degrees of freedom, with an alpha of 0.05, and assuming an attrition rate of 10% for those who (1) withdraw voluntarily from the study, (2) initiate on PrEP but withdraw due to adverse events (e.g. hepatic or renal abnormalities), or (3) become ineligible for PrEP (e.g. seroconvert). The effect size estimate was informed by prior experience from the field, where an effect size of 0.30 has been found between ART clients receiving some adherence support and those receiving none. However, we use a more conservative effect size of 0.15, to account for the fact that adherence support may not lead to the same gains in adherence among HIV-negative PrEP clients as it does with HIV-positive ART clients. The sample size was calculated to be 160 per arm (480 total).

### Primary and secondary outcomes

For Aim 1, our primary outcome will be the proportion of eligible individuals accepting PrEP as part of CBCT services. Secondary outcomes will include (1) stratification of PrEP initiation by both location and CBCT platform; (2) in-depth qualitative understanding of PrEP initiation; (3) correlates of immediate, delayed, and no PrEP initiation; and (4) comparison of sexual behaviours, risk profiles, and self-perception of risk in immediate, delayed, and never acceptors. Using the IMB model to inform the selection of variables and contextualization of results, an assessment of determinants of PrEP uptake will include (1) socio-demographic factors, (2) risk behaviours, (3) self-perception of risk, (4) previous knowledge of PrEP and perception of those taking PrEP, (5) partner characteristics and HIV status, (6) HIV-related stigma, and (7) screening scores for depression, substance abuse, and social support. Baseline STIs will be used as an independent marker of sexual risk behaviour, and to validate self-reported sexual risk behaviours and self-perception of risk.

For Aim 2, the primary outcome will be a comparison of TFV-DP levels by study arm at months 3, 6, 12, 18, and 24, with a 12-month primary outcome and 24-month assessment of intervention durability. Based on cumulative adherence behaviour, as measured in iPrEX OLE [14], adherence will be categorized as (1) *low adherence* (≤ 3 tablets per week; ≤ 699 fmol/DBS punch), and (2) *high adherence* (4–7 tablets per week; ≥ 700 fmol/DBS punch). Analysis of primary outcomes will be conducted on an intention-to-treat basis. Secondary outcomes will include (1) predictors of PrEP adherence after adjusting for study arm, socio-demographic factors, exposure to adherence support activities, and risk profiles; (2) characterization of changes in sexual behaviours and risk profiles following PrEP initiation; (3) proportion of individuals who discontinue PrEP and factors associated with discontinuation; (4) proportion of individuals who discontinue but subsequently restart PrEP and characteristics associated with this subgroup; (5) factors associated with of patterns of PrEP use; and (6) profiles of individuals that may benefit more from group-based vs. individualized adherence support.

For Aim 3, we will estimate incremental costs relative to standard practice for the intervention. Micro-costing studies will use activity-based approaches for costs incurred (e.g. start-up activities, recruitment, service delivery, lab monitoring, adherence support, and PrEP) and costs averted (e.g. health costs saved by averting HIV infections). Adjusting for time spent on research activities (e.g. informed consent, research questionnaires), the total time required for the intervention will be estimated. Time and costs for PrEP delivery through community-based platforms will be estimated through staff interviews, accounting for time available for the intervention. Costs incurred by patients to access PrEP will be assessed through participant interviews. Furthermore, we will estimate the impact of PrEP strategies by measuring: (1) the difference in HIV incident cases among intervention and control arm participants, (2) changes in HIV incident cases at the population level, and (3) changes in disability-adjusted life years (DALYs) between intervention and control arm participants. The model simulates intervention impact and projects the effect on health outcomes over 10 years. Sensitivity analyses will explore the impact of PrEP uptake and adherence on model outcomes outside a study setting, reduction in HIV transmission due to ART adherence, and PrEP supply-chain interruptions.

### Data analysis

Descriptive statistics (e.g. means, standard deviations/variances, medians with inter-quartile ranges, and frequency distributions) will be used to describe participant demographic characteristics. The chi-squared statistic test will be used for categorical variables whereas T-test (parametric) or Wilcoxon rank sum test (non-parametric) will be applied for continuous variables. Transformations of outcome variables will be explored and performed if appropriate. Multivariate logistic regression models will be performed to examine associations between covariates. Since data is collected on one subject over time, the data will be treated as longitudinal data. Generalized linear mixed effect models will be fitted to determine associations of interest with time and intervention effects included as fixed effects and allowing for subject-specific random effects. Alternatively, generalized estimating equations can be used to account for any correlations of repeated data measures, when assessing determinants of PrEP adherence at time points provided we have balanced data with ignorable missing patterns. Time-to-event analysis will be conducted using Kaplan-Meier plots. To determine possible associations with covariates, a Cox-proportional hazard will be used. If the proportional hazard assumption does not hold, an accelerated failure time model will be used. Missing data due to loss to follow-up is inadvertent and inevitable. The extent of missing data will be assessed in the primary analysis. The primary analysis will be conducted under the assumption of data Missing at Random (MAR). As such, longitudinal likelihood-based data analysis, utilizing all the observed pre-deviation data from each participant will be employed. Thereafter, multiple imputation (MI) method will be employed under the primary MAR assumption, and Rubin’s rule will be followed to combine results. Sensitivity to deviation from the MAR assumption will be investigated. Intention to treat (ITT) assessment with complete case approaches where necessary will be implemented in cases of non-adherence to randomization assignment. Depending on the extent of non-adherence, the “as treated (AT)” approaches may be employed to analyse participants according to the intervention received regardless to their randomized allocation. A marker of level of adherence can also be included in the models. Patterns of PrEP use will be identified using latent class models or clustering techniques and the association of factors with these latent classes will be examined using multinomial logistic regression modelling. For all statistical investigations, tests for significance will be two-tailed. Analyses will be conducted using STATA 13.0 or R version 4.

Qualitative data analyses of participant and staff IDIs, CAB FGDs, CMEs, and staff SEPs will be conducted using a constant comparison approach guided by the study IMB framework. Data and results will be triangulated using matrices to refine findings [61]. Transcripts will be analysed by the qualitative research team, with results presented to study leadership for review and to guide additional analyses. Socio-behavioural factors and contextual (urban v. rural) influences influencing young women’s action plans for PrEP uptake and adherence will be used to create risk and adherence profiles to inform eventual intervention tailoring and implementation.

### Ethical considerations and trial registration

PrEP has been approved for use by international and domestic governing bodies in South Africa. Written informed consent will be obtained from participants at multiple time points, including prior to baseline questionnaire, PrEP initiation, and any qualitative interviews. The study protocol has received full ethical approval from the University of Cape Town Human Research Ethics Committee (Ref: 289/2018) and has been registered with ClinicalTrials.gov (NCT03977181). Any and all protocol modifications and annual ethics renewal will be submitted to the University of Cape Town Human Research Ethics Committee for approval.

There are no anticipated problems that are detrimental to the participants; however, expected clinical side effects of DIDIVIR include nausea, dizziness, and diarrhoea; study nurses will be trained on the Southern African HIV Clinicians Guidelines for PrEP used to identify and manage side effects [62]. All research staff will be trained to identify, probe for, and report AEs and social harms (SHs). Occurrence of AEs and SHs will be collected at every visit and recorded in REDCap. All AEs and SHs, with detailed study notes, will be internally reviewed by the study team. An AE list will be compiled and reviewed by the study DSMB and reported to University of Cape Town IRB. A study clinician will review all abnormal test results, liaise with local clinic doctors, and has the authority to terminate participants based on clinical opinion. Upon completion of their 2-year follow-up, all participants will be provided a referral to their local clinics for continuation of PrEP services.

### Dissemination plans

As results are available and finalized, they will be analysed for publication in peer-reviewed scientific journals and presentation at relevant scientific conferences; standard authorship guidelines will be followed, and no professional writers will be used. Results will also be presented, orally and in writing, to local (i.e. Buffalo City Metro District Department of Health), provincial (i.e. Eastern Cape Provincial Department of Health, Eastern Cape Provincial AIDS Council), and key national (i.e. South African National Department of Health, South African National HIV Think Tank) stakeholders. Results will be reported back to study CABs, communities and participants, via town hall style meetings and as 1-pager flyers using infographics. Key interim results (i.e. # HIV seroconversions, STI incidence, and pregnancies), implementation experiences, and lessons learned will be shared with district and provincial stakeholders on a bi-annual basis.

## Discussion

The Community PrEP Study is an innovative, mixed-methods study that aims to provide robust insights into (1) the acceptability, feasibility, and uptake of PrEP as a methods for HIV prevention by AGYW when delivered through CBCT platforms, (2) community-based prevention-effective adherence support for AGYW, and (3) the cost-effectiveness of such community-based approaches to delivering PrEP services. Numerous barriers have been described that delay or block AGYW from accessing clinic-based health services, especially reproductive health and HIV testing and prevention services. Consequently, reaching AGYW at scale with HIV prevention services requires delivery platforms outside of clinic-based facilities. Our use of community-based platforms to increase AGYW’s access to PrEP and provide same-day initiation is novel. If found to be acceptable and feasible, CBCT platforms can be used to target PrEP services to other at-risk and key populations.

Recent work has highlighted that positive peer support [63–65] and cognitive-behavioural problem-solving [46, 66] consistently improve medication adherence among adults and adolescents. Peer norms play an important role in shaping behaviours, especially for adolescents and young people [67, 68], and community-based approaches that build on peer support may have a strong influence on young women. Our group-based health clubs are highly innovative because they will (1) draw on peer support and cohesion to promote prevention-effective adherence to PrEP to prevent HIV infection, (2) provide a forum for adherence support outside the formal clinic environment, (3) create a positive group norm around the use of PrEP, and (4) provide a space to build social relationships and bonds with other AGYW who are using PrEP. If found to be effective, our prevention-effective adherence support interventions may be tailor for other at-risk groups and communities. Finally, given South Africa’s embrace of group-based ART adherence programmes [69, 70], our adherence support intervention will be well positioned to be integrated into South Africa’s current adherence support strategies.

Our study has both benefits and limitations of note. Many PrEP studies involving AGYW have required participants to be sexually active and/or “at-risk” [18–20, 71, 72]. By eliminating these inclusion criteria, we may be able to extend PrEP services and study involvement to AGYW that provide socially desirable answers to sexual activity and risk behaviour questions. Another benefit will be the provision of regular HIV and STI testing, and hepatitis B vaccination services. Though similar services are provided by local health clinics, participants may perceive or truly experience diminished barriers due to the nature and positioning of our community-based services. Of concern, by randomizing participants to a specific support modality, we may be overlooking the specific and/or unique needs and life-styles of individuals. As such, poor efficacy of an intervention arm may not be the true result of the modality itself, but due to the poor matching of a behavioural intervention to individuals. By conducting in-depth interviews with participants, we expect to assess this potential limitation. Finally, the ability to quickly form health club groups with a critical mass of participants will be impacted by the pace of recruitment. To minimize this potential challenge, study staff will closely monitor recruitment rates and initially create smaller groups that can be merged over time to ensure group critical mass. Moreover, given school, work, and familial responsibilities, scheduling of health clubs to ensure consistent attendance by a critical mass of participants may be logistically challenging. To address this, health clubs will be flexibly scheduled to maximize participant attendance.

Findings from our study will provide insight into uptake and prevention-effective use of PrEP to prevent HIV infection by AGYW in both urban and rural contexts. Identifying a prevention-effective adherence intervention suitable for this key population will provide researchers and clinicians with an additional tool integral for the rollout of PrEP. The cost-effectiveness analysis will provide policy makers with the information they need to make evidence-based decisions on the structure and components for a PrEP programme. Finally, the results of our study aim to inform approaches and guidelines related to PrEP programme implementation, rollout, and scale-up in South Africa and other sub-Saharan countries.

### Trial status

This trial was retrospectively registered with ClinicalTrials.gov (NCT03977181) on June 6, 2019. Participant recruitment was initiated on October 22, 2018, with enrolment completion on November 15, 2019. Participant follow-up will continue through November 2022. Protocol version 1.5, 22 May 2019.

## Data Availability

Study investigators (AMM and LGB) will provide the full study protocol, study data, and statistical code and adherence support materials upon reasonable request and approval of an appropriate data sharing agreement.

## Abbreviations

HIV: Human immunodeficiency virus
AGYW: Adolescent girls and young women
PrEP: Pre-exposure prophylaxis
FTC: Emtricitabine
TDF: Tenofovir
STI: Sexually transmitted infections
CBCT: Community-based HIV counselling and testing
CAB: Community advisory boards
BCM: Buffalo City Metro
IMB: Information-Motivation-Behavioural Skills Model of Behaviour Change
REDCap: Research Electronic Data Capture
ACASI: Audio computer-assisted self-interviewing
PEP: Post-exposure prophylaxis
IDI: In-depth interviews
CME: Community mapping exercises
FGD: Focus group discussion
SEP: Staff exchange programmes
HBsAg: Hepatitis B antigen
eGRF: Estimated glomerular filtration
DBS: Dried blood spot
DALY: Disability-adjusted life years
AE: Adverse events
SH: Social harms
CT: *Chlamydia trachomatis*
NG: *Neisseria gonorrhoeae*
TV: *Trichomonas vaginalis*
